# Stabilisation of hollow colloidal TiO_2_ particles by partial coating with evenly distributed lobes†

**DOI:** 10.1039/d0sm02100h

**Published:** 2021-01-13

**Authors:** Bo Peng, Yanyan Liu, Dirk G. A. L. Aarts, Roel P. A. Dullens

**Affiliations:** Department of Chemistry, Physical and Theoretical Chemistry Laboratory, University of Oxford South Parks Road Oxford OX1 3QZ UK roel.dullens@chem.ox.ac.uk; Department of Applied Physics, Aalto University Espoo FI-00076 Finland pengbo006@gmail.com

## Abstract

Photo-catalytically active crystalline TiO_2_ has attracted special attention due to its relevance for renewable energy and is typically obtained by the calcination of amorphous TiO_2_. However, stabilising hollow colloidal TiO_2_ particles against aggregation during calcination without compromising their photocatalytic activity poses two conflicting demands: to be stable their surface needs to be coated, while efficient photocatalysis requires an exposed TiO_2_ surface. Here, this incompatibility is resolved by partially coating TiO_2_ shells with evenly distributed 3-trimethoxysilyl propyl methacrylate (TPM) lobes. These lobes act both as steric barriers and surface charge enhancers that efficiently stabilise the TiO_2_ shells against aggregation during calcination. The morphology of the TPM lobes and their coverage, and the associated particle stability during the calcination-induced TiO_2_ crystallization, can be controlled by the pH and the contact angle between TPM and TiO_2_. The crystal structure and the grain size of the coated TiO_2_ shells are controlled by varying the calcination temperature, which allows tuning their photocatalytic activity. Finally, the durable photocatalytic activity over many usage cycles of the coated TiO_2_ compared to uncoated shells is demonstrated in a simple way by measuring the photo-degradation of a fluorescent dye. Our approach offers a general strategy for stabilising colloidal materials, without compromising access to their active surfaces.

## Abstract 2

A simple synthetic strategy is developed to stabilise hollow colloidal TiO_2_ particles against aggregation during their calcination and their use as the photocatalysts by partially coating the shells with evenly distributed lobes.

## Graphical abstract

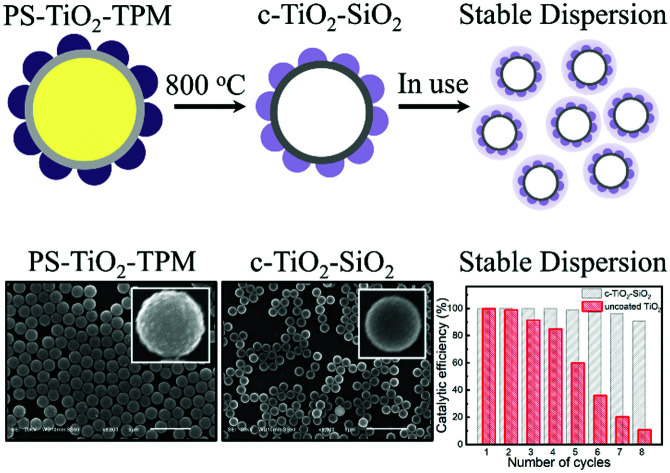

## 1 Introduction

Titanium dioxide (TiO_2_) has been one of the most intensely studied semiconductors over the past few decades due to its wide variety of applications across many different fields,^1–4^ including energy harvesting and storage, photocatalysis, electrochromic devices, sensors, bio-separation, pigment and photonic crystals.^1,2,4–7^ In particular, its application as a photocatalyst in for example water splitting attracted special attention due to its relevance for renewable energy and environmental pollution.^2,4–9^ The photocatalytic activity of TiO_2_ relies on its crystal structure, which sensitively depends on the synthetic strategy adopted.^1,6^ Crystalline hollow colloidal TiO_2_ particles are preferable due to their large active surface area and are conventionally prepared by combining sol–gel, template-sacrifice and calcination approaches.^10–18^ During calcination, the photocatalytically inactive amorphous TiO_2_ is transformed into photoactive crystalline TiO_2_, where the exact crystal form depends on the calcination temperature.^1^ However, calcination induces simultaneous structural rearrangement and grain growth in the colloidal TiO_2_ particles, which makes aggregation between them often unavoidable.^13,19,20^

Recently, a ‘wrap-bake-peel’ strategy has been proposed to overcome these problems, which involves growing a homogeneous silica (SiO_2_) shell around the material of interest to prevent particle aggregation during calcination, and then removing this SiO_2_ shell to expose the ‘active’ surface.^21^ Further progress has been made by selecting different materials for the core and the protective shells.^19,22,23^ However, in all of these approaches the homogeneous shells had to be removed after calcination to expose the ‘active’ surface of the core material. Unfortunately, the removal of the shells compromises the stability of crystalline TiO_2_ shells in a practical environment such as wastewater, where the presence of ions and polymers screens the electric double layer^24^ and induces depletion attractions,^25^ respectively. The stability of these particles in such contaminated aqueous media is further deteriorated by the relatively strong van der Waals attractions between the high refractive index TiO_2_ shells.^26–29^ Thus, the preparation of stable photocatalytic TiO_2_ shells is a serious challenge because photocatalysis requires an exposed active surface, while a protective coating is needed for their stabilization during calcination and subsequent usage as a photocatalyst in aqueous environments.

Here, we overcome this challenge by partially coating colloidal TiO_2_ shells with evenly distributed lobes that stabilize the shells during their calcination and their subsequent use as photocatalysts. To this end, the TiO_2_ shells are covered by evenly distributed TPM (3-trimethoxysilyl propyl methacrylate) droplets, which are polymerized into solid lobes.^30,31^ Conveniently, the morphology of these protective lobes on the TiO_2_ shells can be tuned by varying the pH and the contact angle between TPM and TiO_2_. In addition, the crystal structure and the grain size of the crystalline TiO_2_ shells can be controlled by the calcination temperature. Finally, we demonstrate the photocatalytic activity of our partially coated colloidal TiO_2_ shells in a simple way by measuring the decomposition of a fluorescent dye in water and show that they exhibit photocatalytic activity over many usage cycles compared to uncoated TiO_2_ shells.

## 2 Materials and methods

### 2.1 Materials

All chemicals are used as received from Sigma-Aldrich unless mentioned otherwise. For the synthesis of cationic polystyrene (PS) particles, styrene and 2-(methacryloyl)ethyltrimethylammonium chloride (MTC, 80 wt% in H_2_O) are used as the monomers with the initiator azobisisobutyronitrile (AIBN), recrystallized from ethanol. Polyvinylpyrrolidone (PVP-40) and ethanol serve as the stabilizer and co-solvent, respectively. Titanium(iv) tetraisopropoxide (TTIP, 97%) and 3-(trimethoxysilyl)propyl methacrylate (TPM, 98%) are the precursors for synthesising TiO_2_ and TPM shells, respectively. Ammonia (25% in H_2_O) is used as the catalyst during TPM coating. Rhodamine b isothiocyanate (RITC) is employed for the photo-catalytic evaluation experiments. De-ionized water is used throughout the experiments and obtained from a Millipore Direct-Q UV 3 reverse osmosis filter apparatus.

### 2.2 Methods

#### 2.2.1 Preparation of polystyrene cores

**Fig. 1:**
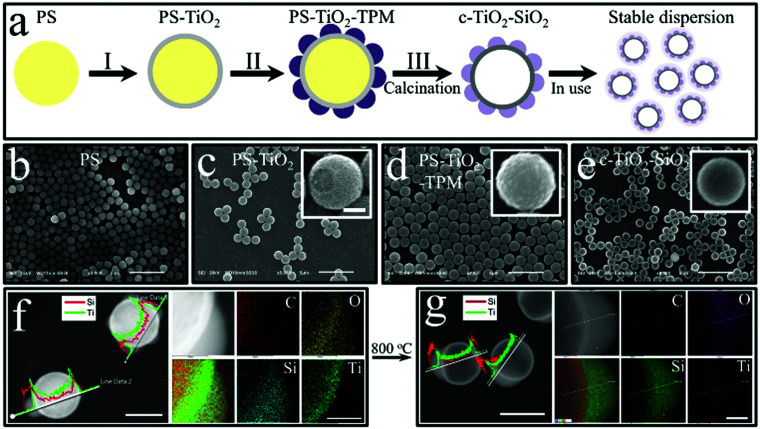
Synthesis and characterization of hollow colloidal TiO_2_ particles partially coated by evenly distributed TPM lobes. (a) A schematic of the preparation of crystalline-TiO_2_–SiO_2_ (c-TiO_2_–SiO_2_) shells *via* three steps: (I) coating cationic polystyrene (PS) with TiO_2_; (II) coating PS–TiO_2_ particles with evenly distributed TPM lobes; (III) calcinating PS–TiO_2_–TPM into c-TiO_2_–SiO_2_. (b–e) Scanning electron microscopy (SEM) observation of (b) PS, (c) PS–TiO_2_, the inset showing a broken TiO_2_ shell, (d) PS–TiO_2_–TPM, and (e) c-TiO_2_–SiO_2_ particles. (f and g) Dark-field transmission electron microscopy images and energy dispersive X-ray spectroscopy with elemental line-scans and map spectra of PS–TiO_2_–TPM and c-TiO_2_–SiO_2_ particles, respectively. The scale bars are 5 μm in (b–e), 500 nm in the inset of (c), which also applies to the insets in (d) and (e), 1 μm in (f and g) and 250 nm in the map insets.

To prepare monodisperse cationic polystyrene (PS) colloidal cores, a co-dispersion polymerization of styrene and MTC is adopted from previous work.^32^ Initially, the ethanol/water mixture (25 g, 4 : 1 in mass) consisting of PVP (2.5 g), styrene (5 g) and AIBN (0.25 g) was fed into a 250 mL, three-necked flask equipped with a gas supply, a condenser, and Teflon-coated magnetic stir bar. This mixture was homogenized with a magnetic stir at 200 rpm and deoxygenized with nitrogen for 30 min prior to heating up to 70 °C to commence the polymerization. In order to not disturb the nucleation stage, a mixture comprising of ethanol (10 g), water (2.5 g), styrene (5 g) and MTC (0.3 g) was continually fed into the flask within 2 h, starting from at the point at which the polymerization had been running for 1 h.^33,34^ The reaction was considered complete in 24 h, and resulted in monodisperse PS cores with a mean diameter of 1.07 μm (see Fig. 1b and Fig. S1a, ESI†). PS cores were washed by repeated centrifugation and redispersion (three times) with ethanol and stored in ethanol with a mass content of 11.1%.

#### 2.2.2 TiO_2_ shell growth

The coating procedure for growing a TiO_2_ shell onto the PS cores is adapted from the methods of Cheng *et al.*^35^ and Imhof,^14^ and carried out in an ethanol environment at room temperature by hydrolysing titanium tetraisopropoxide (TTIP) in the presence of PS microspheres. First, 5 g of PS dispersion was diluted with 100 mL of ethanol consisting of PVP (1 g) and water (1 g). Then, a mixture of TTIP (1 mL) and ethanol (5 mL) was rapidly injected into PS dispersion while vigorously stirring at 1000 rpm. After 1 min the stirring was stopped, and the suspension was kept stationary overnight. Finally, the core–shell PS–TiO_2_ particles were washed twice by centrifugation and redispersion in ethanol and twice in water, and finally stored in water (at a concentration of 3.5 wt%) for the next step.

#### 2.2.3 Coating with TPM lobes

Coating the PS–TiO_2_ particles with TPM lobes is a three-step procedure: pre-hydrolysis, coating and solidification. Certain experimental parameters can be varied to alter the morphology of the TPM coatings. A typical TPM coating is carried out as follows: firstly, a solution of pre-hydrolysed TPM was prepared by mixing 1 mL of TPM with 19 mL of H_2_SO_4_ aqueous solution (0.1 mM) under magnetic stirring at 1000 rpm until the solution becomes transparent (<4 h).^30,31^ The pre-hydrolysed TPM solution should be used freshly or stored at 4 °C less than 24 h. Secondly, 0.5 mL of PS–TiO_2_ aqueous dispersion was mixed with 19.5 mL of PVP aqueous solution and 100 μL of ammonia by stirring at 400 rpm for 2 h in a 30 mL glass vial. Then, 1 mL of pre-hydrolysed TPM was added to the PS–TiO_2_ suspension, and the mixture was gently shaken by hand for 30 s every 10 min. After 1 h, 20 mg of AIBN was added into the mixture. The hydrolysed TPM was gradually condensed and precipitated from water in the form of droplets. Thirdly, these liquid TPM droplets were polymerized by placing the vial in the oven at 80 °C overnight. After the synthesis, the PS–TiO_2_–TPM particles were collected by centrifugation and washed three times by centrifugation and redispersion in water. This synthesis is scalable in volume as exemplified in Fig. S2 (ESI†), where the original recipe was scaled up by 200 times.

#### 2.2.4 Calcination

The composite PS–TiO_2_–TPM particles were dried to a powder overnight under an N_2_ stream at room temperature and put into alumina crucibles with caps to prevent contamination. Placing samples into a furnace at 500 °C for 5 min, and then increasing the temperature to the target temperature within 5 min can gradually crystallize amorphous TiO_2_ into its anatase and further to rutile crystals mainly depending on the temperature and TPM coating. At the same time, TPM was converted to SiO_2_ and PS was decomposed. After 1 h calcination, the crucibles were withdrawn and cooled down naturally to room temperature.

#### 2.2.5 NaOH etch

Converting TPM into SiO_2_ by calcination offers a possibility to remove the lobes with NaOH. To this end, the PS–TiO_2_–SiO_2_ particles were mixed with NaOH aqueous solution (0.5 M) in a plastic bottle by magnetically stirring at 400 rpm overnight. The solid residue was collected by centrifugation and washed with water by centrifugation until the pH was about 7.

#### 2.2.6 Photocatalysis characterization

Typically, 5 mg of particles was dispersed into 40 mL deionized water by sonication. Prior to the photocatalytic experiments, the particle dispersion was irradiated under a UV lamp (Hamamatsu lightningcure LC8, 410 mW cm^−2^ at 365 nm) at a distance of 10 cm for 30 min to rule out the influence of any organic residues in the dispersion. Then, 10 mL of RITC (0.1 mM) aqueous solution was added into the dispersion. After stirring in the dark for 30 min to ensure the surface of the particles has been saturated with the dye, the UV light was switched on. During the illumination, samples were taken out from the solution at 10 min intervals. The particles were sedimented by centrifugation, and the supernatants were stored in the dark prior to UV-Vis measurements. In the recyclability tests, the particles were kept in their dispersions throughout the whole experiment. For each cycle, a 1 mL of RITC (1 mM) aqueous solution was added into the particle suspension at the beginning of the photodegradation reaction, and the extra UV irradiation was applied until the dye were completely degraded to prevent any dye residue prior to next cycle. Note that the samples taken for UV-Vis measurements were fed back to the particle dispersion to avoid the loss of any particles.

#### 2.2.7 Particle characterization

Scanning electron microscopy (SEM, JEOL JSM-6010LV) was used to determine the size and polydispersity of the TPM lobes, but also to investigate the surface morphology of the particles. Prior to observation, samples were dried on a thin silicon wafer that was adhered to a SEM stub, and then sputter-coated with a layer of platinum/gold. The number-averaged sizes (*D*) of the particles and lobes, and their standard deviations (*σ*) were obtained by measuring more than 100 particles. The polydispersity (*δ*) of the particles is defined as *δ* = *σ*/*D*. To characterize the internal structure and elemental distribution of the samples, a JEOL JEM-2100 transmission electron microscope (TEM) equipped with energy-dispersive X-ray spectroscopy (EDX) was performed at an accelerating voltage of 200 kV. Samples suitable for TEM observation were prepared by casting a droplet of diluted particle dispersion onto the carbon-coated copper grids and then drying at room temperature. The stability of the samples was recorded with an Ximea camera (USB 3.0) mounted on an Olympus IX73 optical microscope.

The crystalline structures of the samples were evaluated with a PANalytical X’Pert Pro X-ray diffractometer (XRD) equipped with a monochromatized Cu Kα X-ray source (*λ* = 1.5403 Å). The grain size of the samples was estimated with Debye–Scherrer equation, *τ* = *Kλ*/*β* cos *θ*, where τ is the mean size of the grain; *K* the dimensionless shape factor, in this case, 0.89; *λ* the wavelength of the X-ray source; *β* the width of the XRD peak at the half-peak height in radians, and *θ* the Bragg angle. The thermogravimetric analysis (TGA) and differential scanning calorimetry (DSC) were performed on a Mettler Toledo TGA/DSC 1 system by heating up the sample from room temperature to 1000 °C with a rate of 5 °C/min in air. The Zeta potential of the samples was measured with a Malvern Zetasizer Nano.

## 3. Results and discussion

The three-step preparation of the partially coated colloidal TiO_2_ shells is schematically shown in Fig. 1a. First, monodisperse cationic polystyrene (PS) particles with a mean diameter of 1.07 μm (see Fig. 1b, Fig. S1a, and Table S1, ESI†) are synthesized by dispersion polymerization^32,34^ (for details see Section 2.2.1 and Fig. S1, Table S1, ESI†). Next, the cationic PS particles are coated with an anionic TiO_2_ shell of 50 nm^14,35^ (step I in Fig. 1a, see also Fig. 1c and Fig. S1b, Table S1, ESI†). Then, the PS–TiO_2_ particles are mixed with pre-hydrolysed TPM in alkaline water in the presence of the stabilizer polyvinylpyrrolidone (PVP), which results in raspberry-like PS–TiO_2_–TPM particles, as shown in Fig. 1d (step II, which can be scaled up in volume, see Fig. S2, ESI†). Finally, the PS–TiO_2_–TPM particles are calcinated at a temperature within a range of 500–1000 °C (step III). Importantly, during calcination the PS core is removed, the TPM is converted into SiO_2_ and the initially amorphous TiO_2_ crystallizes, which eventually results in hollow colloidal TiO_2_ particles partially coated with evenly distributed SiO_2_ lobes. In the following we will denote these particles as crystalline-TiO_2_–SiO_2_, or in short as c-TiO_2_–SiO_2_ as shown in Fig. 1e. The composition of the particles before (PS–TiO_2_–TPM) and after (c-TiO_2_–SiO_2_) calcination is characterized by energy dispersive X-ray spectroscopy with elemental line-scans and map spectra as displayed in Fig. 1f and g. Further characterization with transmission electron microscopy and energy dispersive X-ray spectroscopy with elemental analysis in a selected area, and electron diffraction is shown in Fig. S3 (ESI†). These analyses indeed confirm the presence of TiO_2_ and its crystallization, the conversion of the TPM lobes into silica, and the thermal decomposition of the PS cores.

**Fig. 2:**
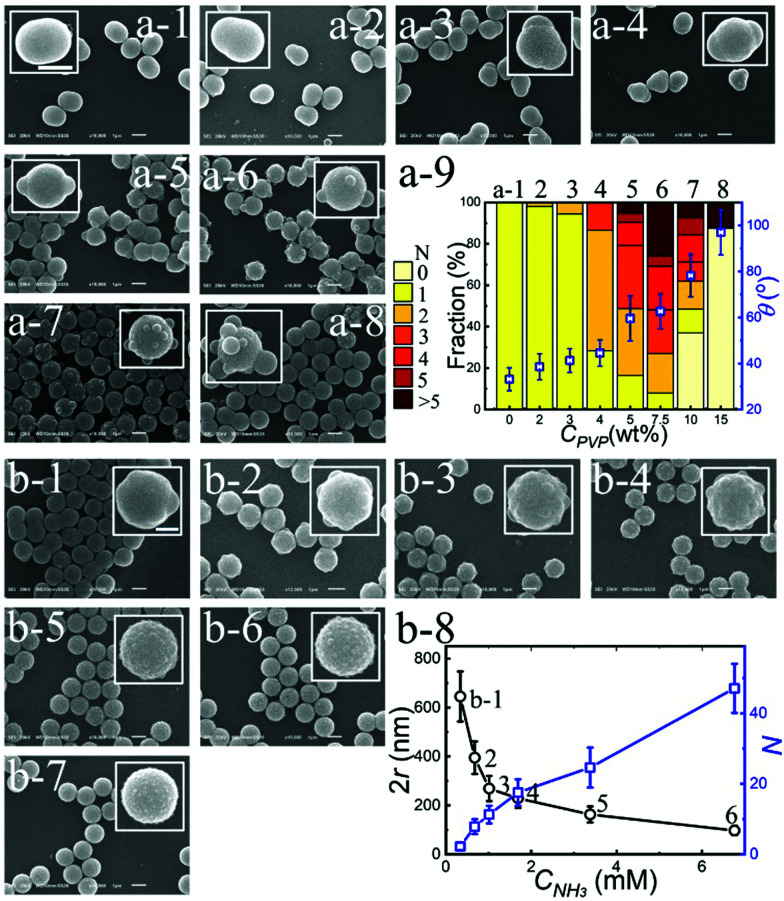
Controlling the surface morphology of TPM lobes. SEM images of TPM coated TiO_2_ particles at increasing PVP concentration (*C*_PVP_): (a-1) 0, (a-2) 2, (a-3) 3, (a-4) 4, (a-5) 5, (a-6) 7.5, (a-7) 10, to (a-8) 15 wt%, respectively; (a-9) a graphic summary of *C*_PVP_ effect on the number of TPM lobes (*N*) and the contact angle (*θ*). Controlling the surface roughness by varying the ammonia concentration (*C*_NH_3__) at a *C*_PVP_ of 5 wt%: (b-1) 0.338, (b-2) 0.676, (b-3) 1.014, (b-4) 1.69, (b-5) 3.38, (b-6) 6.78, to (b-7) 16.9 mM, respectively; (b-8) The lobe size (2*r*) and *N* as a function of *C*_NH_3__, where the error bars indicate the polydispersity (for details see ESI,† Section S1.2.7). Note that the data corresponding to (b-7) are not included in (b-8) because the lobe size in (b-7) is too small to be reliably measured. *N* is measured from SEM images. Scale bars are 1 μm and error bars are standard deviation.

The surface morphology of the evenly distributed TPM lobes that partially coat the TiO_2_ shell, is of crucial importance for the particle stability during the calcination and its subsequent application as a photocatalyst (see Fig. 1a, final panel). The morphology of the TPM lobes on the TiO_2_ surface can be quantified using the following three geometric parameters: the number of lobes (*N*), the contact angle between the TPM lobe and the TiO_2_ surface (*θ*) and the lobe size (*r*) as defined in Fig. S4 (ESI†). Conveniently, all these parameters can be tuned by varying the experimental conditions. In particular, increasing the PVP concentration (*C*_PVP_) increases both *N* and *θ*, as shown in Fig. 2a. As the contact angle increases, the coalescence of liquid TPM droplets on the TiO_2_ surface is suppressed, resulting in an increasing fraction of particles with a larger number of lobes (*N*). At very high PVP concentrations (*C*_PVP_ > 7.5 wt%), however, the attachment of TPM lobes becomes very unfavourable as is evident from the small fraction of particles with *N* > 0 (Fig. 2a-9). This, in fact, is reminiscent of grime-removal with detergents at colloidal scales, where PVP acts as the detergent, breaking up and removing TPM lobes from PS–TiO_2_ colloids. Based on these observations, we have used a *C*_PVP_ of 5 wt% in the following experiments (Fig. 2b).

The surface morphology of the TPM lobes can be further tuned by varying the concentration of the catalyst NH_3_ (*C*_NH_3__). As illustrated in Fig. 2b-1–b-7, the lobe radius *r* decreases while the number of lobes *N* increases with increasing *C*_NH_3__, *i.e.*, increasing pH. Ammonia catalyses the condensation of pre-hydrolysed TPM into droplets. Hence, increasing *C*_NH_3__ leads to the precipitation of many small-sized TPM lobes on the TiO_2_ surface, as demonstrated in Fig. 2b-8. Another efficient way to tune the TPM surface morphology is to alter the amount of pre-hydrolysed TPM, as shown in Fig. S5 and S6 (ESI†). Here it is observed that the lobe size increases with the amount of added hydrolysed TPM. We roughly estimate the maximum surface coverage to be about 60%. The fact that *N* decreases at the same time, suggests that the lobes may be coalescing.

**Fig. 3:**
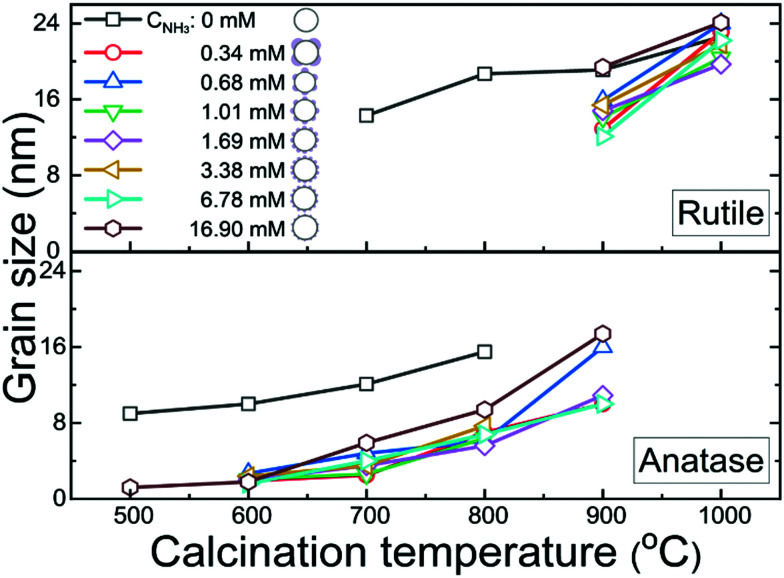
The grain size dependence on the calcination temperature for the anatase and rutile TiO_2_ crystal phases, as determined from X-ray diffraction spectra (detailed in Fig. S7 and Table S2, ESI†), for different ammonia concentrations, *C*_NH_3__, used during the synthesis (corresponding to the samples shown in Fig. 2b-1–b-8). Note that the samples with *C*_NH_3__ = 0 are uncoated pristine TiO_2_ shells and the other samples are c-TiO_2_–SiO_2_ shells.

Calcination is one of the key process in the production of photo-catalytically active TiO_2_, as this transforms amorphous TiO_2_, which is commonly regarded as being photo-catalytically inert, into crystalline and photo-catalytically active TiO_2_ phases.^2,15^ Here, both TiO_2_ shells coated with TPM lobes and uncoated TiO_2_ shells were exposed to calcination temperatures between 500–1000 °C. As shown in Fig. 3 and Fig. S7, Table S2 (ESI†), for the uncoated TiO_2_ shells the rutile phase appears at ∼700 °C. In contrast, for the coated TiO_2_ shells the anatase phase^5,20^ emerges at ∼500–600 °C, while the photo-catalytically less active rutile phase only appears at ∼900 °C. This is also verified by differential scanning calorimetry (see Fig. S8, ESI†), as well as transmission electron microscopy and electron diffraction of a selected area (see Fig. S3e–g, ESI†). Coating the TiO_2_ shells with TPM lobes thus widens the thermal stability range of the photo-catalytically more active anatase phase.

Coating the TiO_2_ shells with TPM lobes also affects the grain size and stability during calcination. As seen in Fig. 3, the grain size increases with increasing calcination temperature for both the coated and uncoated TiO_2_ shells (Fig. S7 and Table S2, ESI†), though it is smaller for coated shells. We speculate that this is due to the water-soluble silicate being absorbed in the small pores of the TiO_2_ shell, which would limit the grain growth of TiO_2_ during calcination^20^ The overlap of the data for different ammonia concentrations (*C*_NH_3__) in Fig. 3 suggests that the grain size and the relative stability of the anatase and rutile phases are not affected by the surface morphology of the TPM lobes, which is controlled by the *C*_NH_3__ during the synthesis in step II (see Fig. 2b). Importantly, however, the presence of the TPM lobes does significantly enhance the stability of the TiO_2_ shells during calcination. While the hollow morphology of the coated TiO_2_ shells and their stability are retained during calcination (see Fig. S9–S15, ESI†), the uncoated TiO_2_ shells collapse and aggregate, especially for calcination temperatures above 600 °C (see Fig. S16, ESI†). In particular, TiO_2_ shells coated with evenly distributed and moderately sized (∼100 nm) TPM lobes (Fig. S14, ESI† and Fig. 2b-6) survive the calcination up to 1000 °C. TiO_2_ shells that are either sparsely coated with TPM lobes (Fig. S9–S13, ESI,† and Fig. 2b-1–b-5) or with very small lobes (Fig. S15, ESI† and Fig. 2b-7) aggregate during calcination.

**Fig. 4:**
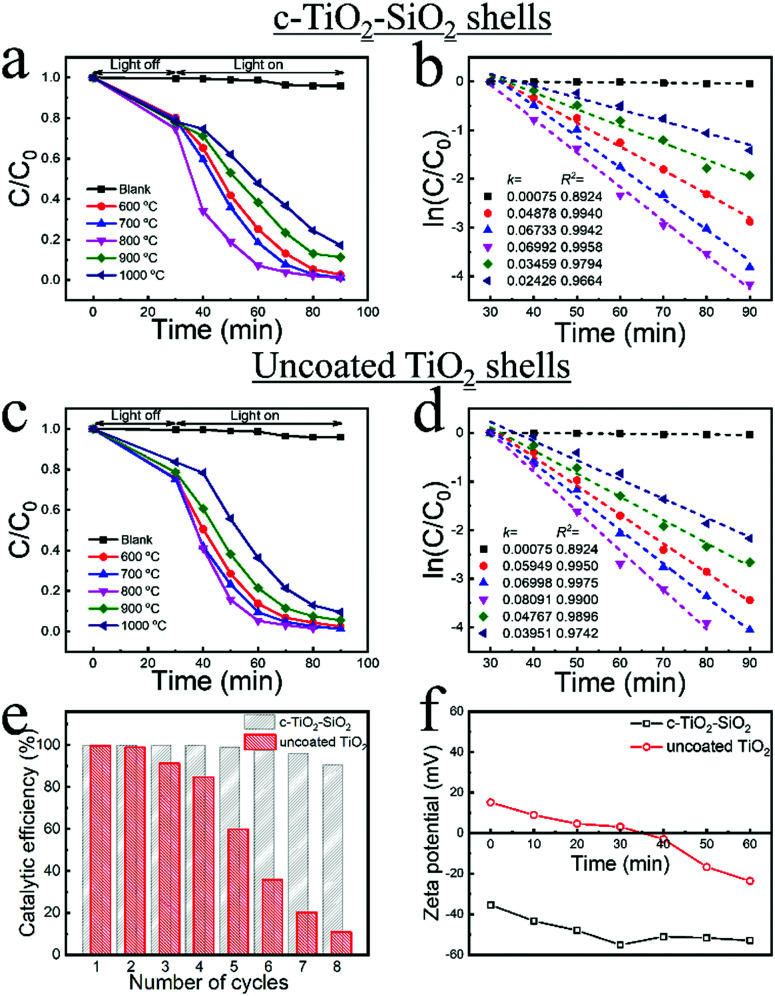
The photocatalytic activity and stability of c-TiO_2_–SiO_2_ and uncoated TiO_2_ shells during the degradation of rhodamine b isothiocyanate (RITC) under UV irradiation measured at the wavelength of maximum absorbance of RITC. (a) The relative absorbance (*C*/*C*_0_) as a function of the irradiation time using c-TiO_2_–SiO_2_ shells (see Fig. 2b-6 and Fig. S14b–f, ESI†) calcinated at temperatures between 600 to 1000 °C; (b) the corresponding fits to the data in (a) to obtain the effective first order reaction rate constants (*k*); (c and d) the same quantities as in (a and b) for the uncoated shells, which are obtained by removing the SiO_2_ lobes of the coated shells using NaOH. (e) A recycling comparison in catalytic efficiency of c-TiO_2_–SiO_2_ (sample in a) and TiO_2_ (sample in c) shells. (f) The zeta potential as a function of time of c-TiO_2_–SiO_2_ and uncoated TiO_2_ shells during the photocatalysis.

We next compare the photocatalytic activities of the TPM coated TiO_2_ shells, and the uncoated TiO_2_ shells, at different calcination temperatures by measuring the photo-degradation of rhodamine b isothiocyanate (RITC) under UV irradiation^36^ (for details see Section 2.2.6, and Fig. S17, ESI†). RITC is a common dye widely used in polymer and colloidal experiments.^33,37,38^ The catalytic degradation of a dye is a simple, yet convenient experiment for demonstrating the particles’ stability, and we note that the study of the photodegradation mechanism is beyond the focus of this work. In particular, we compare the photo-catalytic activity of TiO_2_ shells coated with evenly distributed lobes (Fig. 4a and b) with that of uncoated TiO_2_ shells (Fig. 4c and d). Note that the uncoated TiO_2_ shells (Fig. S18 and S19, ESI†) used for these experiments were obtained by removing the SiO_2_ lobes of already coated and calcinated shells (c-TiO_2_–SiO_2_ in Fig. S14b–f, ESI†) using NaOH (see Materials and methods, Section 2.2.5). Clearly, the photo-catalytic activities of TiO_2_ shells both depend non-monotonically on the calcination temperature: their catalytic activities exhibit a maximum at 800 °C, with the activity between 600–700 °C being larger than that between 900–1000 °C. Correlating this with the data in Fig. 3, shows that the anatase TiO_2_ is catalytically more active than its rutile form.^5,20^

Although the rate constants (*k*) quantifying the kinetics of the photo-degradation of RITC are ∼20% smaller for the coated TiO_2_ shells, their recycling stability is superior to that of the uncoated TiO_2_, as shown in Fig. 4e. While the catalytic efficiency of coated TiO_2_ shells remains around 90%, only about 10% of the initial catalytic efficiency is retained for uncoated TiO_2_ shells after 8 cycles of re-usage, which we attribute to the significantly increased stability of the coated TiO_2_ shells during their usage as photo-catalyst. This is corroborated by the fact that the uncoated TiO_2_ shells initially are weakly positively charged after NaOH treatment, and their zeta-potential decreases and even changes sign during the catalysis (see Fig. 4f). This decrease and sign-reversal of the surface charge will destabilize and lead to aggregation of the uncoated TiO_2_ shells (Fig. S20, ESI†). The underlying mechanism of the surface charge reversal may be complex with many factors contributing to it including for instance the pH and the different products of the RITC degradation, and should be interesting for further studies. In contrast, the coated TiO_2_ shells are strongly negatively charged, and their zeta-potential initially becomes even more negative during the catalysis before it plateaus, thereby ensuring their stability as the photo-catalysis progresses. As a reference, we also characterized the photo-catalytic activity of pristine TiO_2_ shells, which tend to aggregate during calcination (Fig. S16, ESI†) and exhibit significantly lower photo-activity than both the TPM coated and uncoated TiO_2_ shells calcinated at the same temperature (Fig. S21, ESI†). This corroborates that partially coating the TiO_2_ shells with evenly distributed TPM lobes is an effective way to stabilize colloidal photocatalysts without compromising their photocatalytic activity.

## 4 Conclusions

In summary, we have developed colloidal TiO_2_ shells that are partially covered with evenly distributed TPM lobes, which stabilize the shells during their calcination and their use as photocatalysts. The coverage and morphology of these lobes on the TiO_2_ shells can be tuned by varying the pH and the contact angle between TPM and TiO_2_. In addition, both the grain size and the crystal structure of the crystalline TiO_2_ shells can be tuned *via* the calcination temperature. We have demonstrated the photocatalytic activity of the partially coated colloidal TiO_2_ shells by quantifying the decomposition of a fluorescent dye in water and the TPM coated TiO_2_ shells exhibit photocatalytic activity over many usage cycles compared to uncoated TiO_2_ shells. Our work thus not only represents a new route for synthesising partially coated colloidal TiO_2_ shells with an enhanced and durable stability, but it also provides a general strategy to stabilize colloidal materials while maintaining access to their active surfaces.

## Conflicts of interest

There are no conflicts to declare.

## Supplementary Material

SM-017-D0SM02100H-s001
