# Supplementary material for: Stabilisation of hollow colloidal TiO2 particles by partial coating with evenly distributed lobes
Source: Soft Matter. 2021 Jan 13;17(6):1480–6. doi: 10.1039/d0sm02100h (PMC8778591; doi:10.1039/d0sm02100h)
Supplement: SM-017-D0SM02100H-s001 [file SM-017-D0SM02100H-s001.pdf]

Electronic Supplementary Material (ESI) for Soft Matter.
This journal is © The Royal Society of Chemistry 2021

Electronic Supplementary Information

Stabilisation of hollow colloidal TiO_2 particles by partial coating with evenly distributed lobes

Bo Peng,*[ab] Yanyan Liu,[a] Dirk G. A. L. Aarts[a] and Roel P. A. Dullens*[a]

[a] Department of Chemistry, Physical and Theoretical Chemistry Laboratory, University of Oxford, South Parks Road, Oxford OX1 3QZ, United Kingdom.

[b] Department of Applied Physics, Aalto University, Espoo FI-00076, Finland.

Corresponding authors:

Dr. Bo Peng, E-mail: pengbo006@gmail.com; Telephone: +358(0)50 511 6700

Prof. Roel P. A. Dullens, E-mail: roel.dullens@chem.ox.ac.uk; Telephone: +44(0)1865 275 478

1. Supplementary Figures, Tables, and Calculation Details

1.1 Characterization of PS, $PS-TiO_2$, and $PS-TiO_2$-TPM Particles

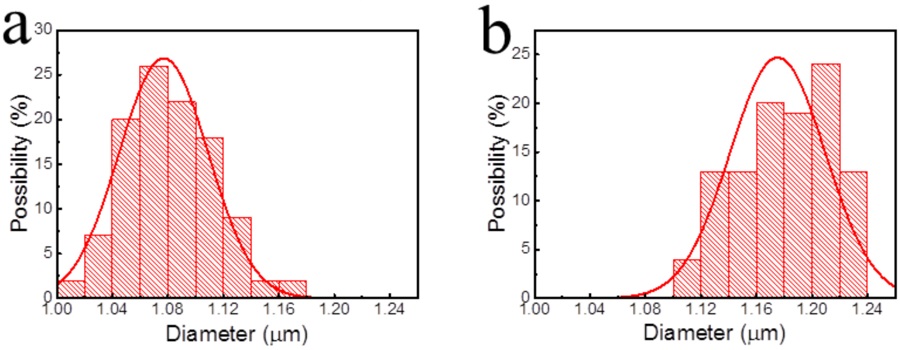

Fig. S1. Size histograms and their normal fits of a) PS cores (corresponds to Fig. 1b), and b) $PS-TiO_2$ particles (Fig. 1c).

Table S1. Size and zeta potential characterization of PS and $PS-TiO_2$ particles.

	PS	$PS-TiO_2$
Mean diameter (μm)	1.07	1.17
Polydispersity (%)	2.9	2.9
Zeta potential (mV)	+16.6	-8.5

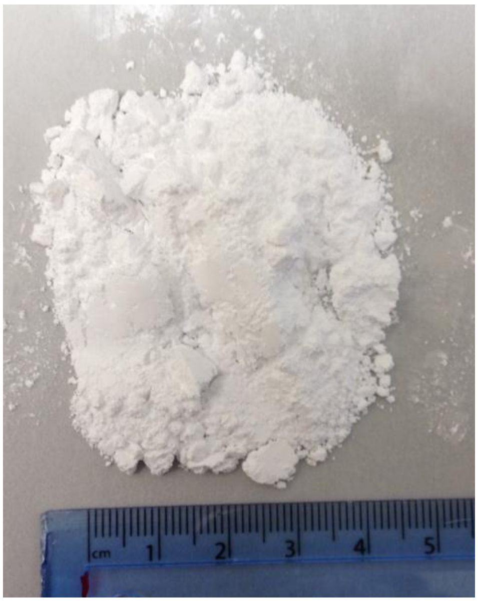

Fig. S2. The powder of $PS-TiO_2$-TPM particles resulting from a scaled-up version of the synthesis. The particles yield was about 4 g.

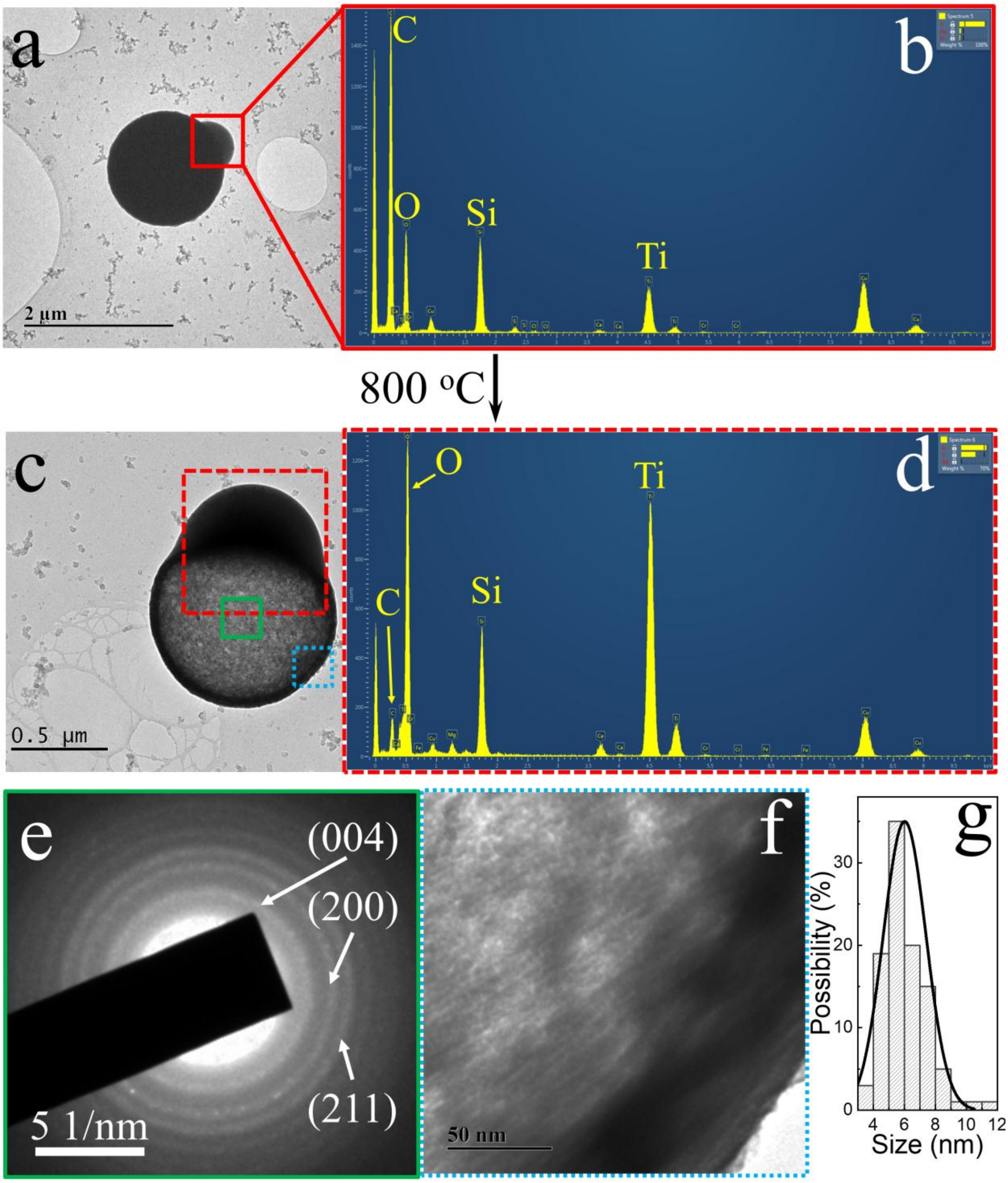

Fig. S3. Characterization of hollow colloidal TiO_2 particles partially coated by TPM lobes. a) Transmission electron microscopy (TEM) image and b) selected area elemental analysis of PS-TiO_2-TPM particles. c) TEM, d) elemental analysis, e) selected area electron diffraction, f) high-resolution TEM, and g) grain size distribution of c-TiO_2-SiO_2 particles. After the calcination at 800 ^{o}C, the particles became hollow as shown in a) and c), and the abundance of C significantly decreased as can be inferred from comparing b) and d). This is ascribed to the thermal decomposition of PS and the transformation of TPM into SiO_2. In addition, the TiO_2 was annealed into poly-anatase crystals with a mean grain size of 6.02 nm as shown in e), f), and g). These results are consistent with the data shown in Figs. 1 and 3.

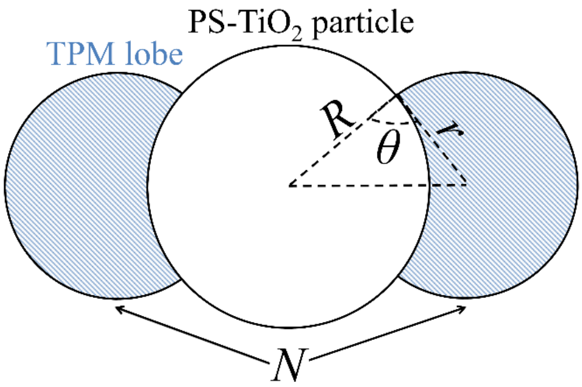

Fig. S4. Schematic model of the PS-TiO_2-TPM particles. *R*, *r*, *θ*, and *N* are the averaged radius of the PS-TiO_2 particles, the radius of TPM lobes, contact angle, and lobe number, respectively.

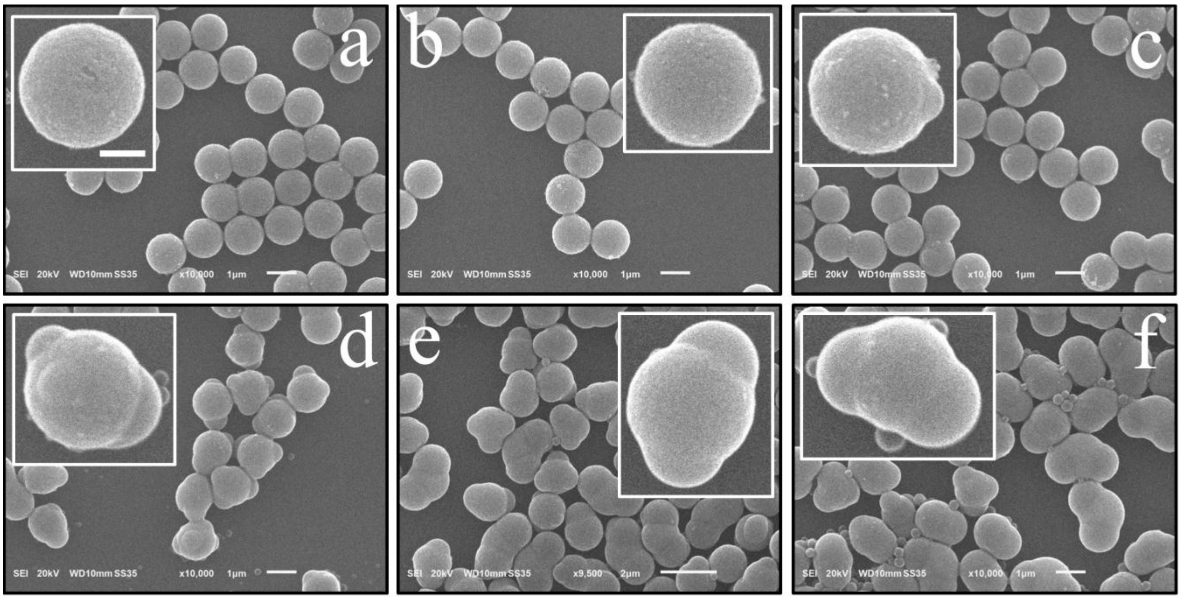

Fig. S5. Scanning electron microscopy (SEM) images of TPM coatings on PS-TiO_2 particles. The volumes of pre-hydrolysed TPM used are 0.3, 0.5, 0.7, 1.5, 2.0 and 3.0 ml in a)-f), respectively, with a C_{NH_3} of 0.676 mM and C_{PVP} of 4.0 wt%. The scale bar is 500 nm in the insets. Increasing the volume of pre-hydrolysed TPM leads to an increase in *r* and the surface coverage of the shells and a decrease in *N*.

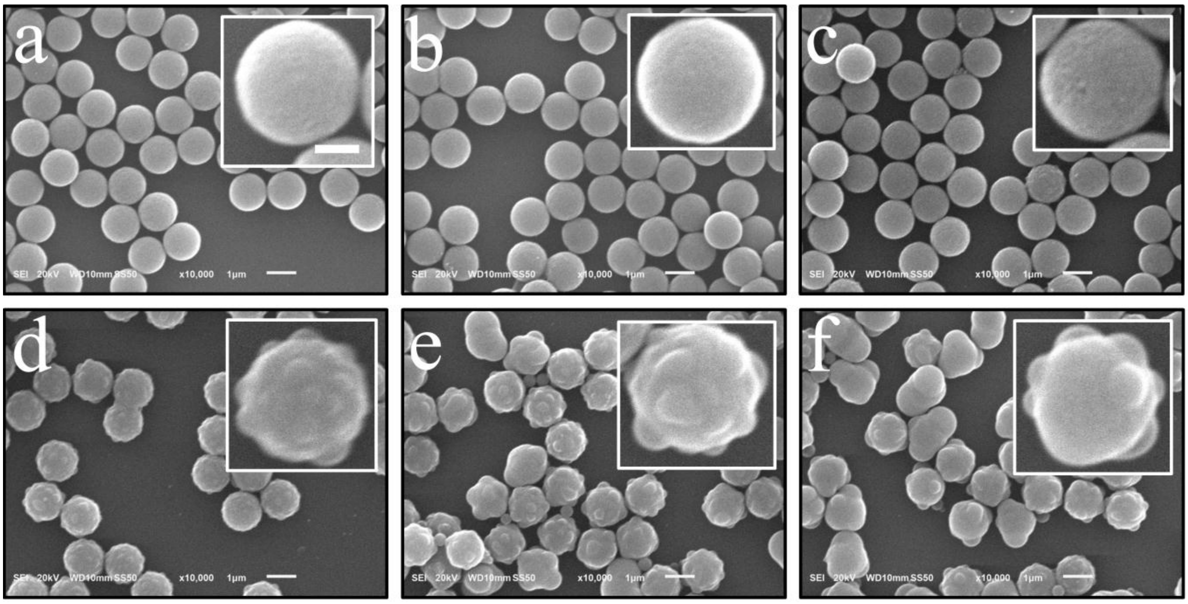

Fig. S6. SEM images of PS-TiO_2-TPM particles for different volumes of pre-hydrolysed TPM used: 0.3, 0.5, 0.7, 2.0 and 3.0 ml in a-f), respectively, with a C_{NH_3} of 3.38 mM and C_{PVP} of 4.0 wt%. The scale bars are 1 μm and 500 nm in the insets, respectively. Increasing the volume of pre-hydrolysed TPM leads to an increase in *r* and the surface coverage of the shells and a decrease in *N*.

1.2 Characterization of calcinated PS-TiO_2-TPM particles

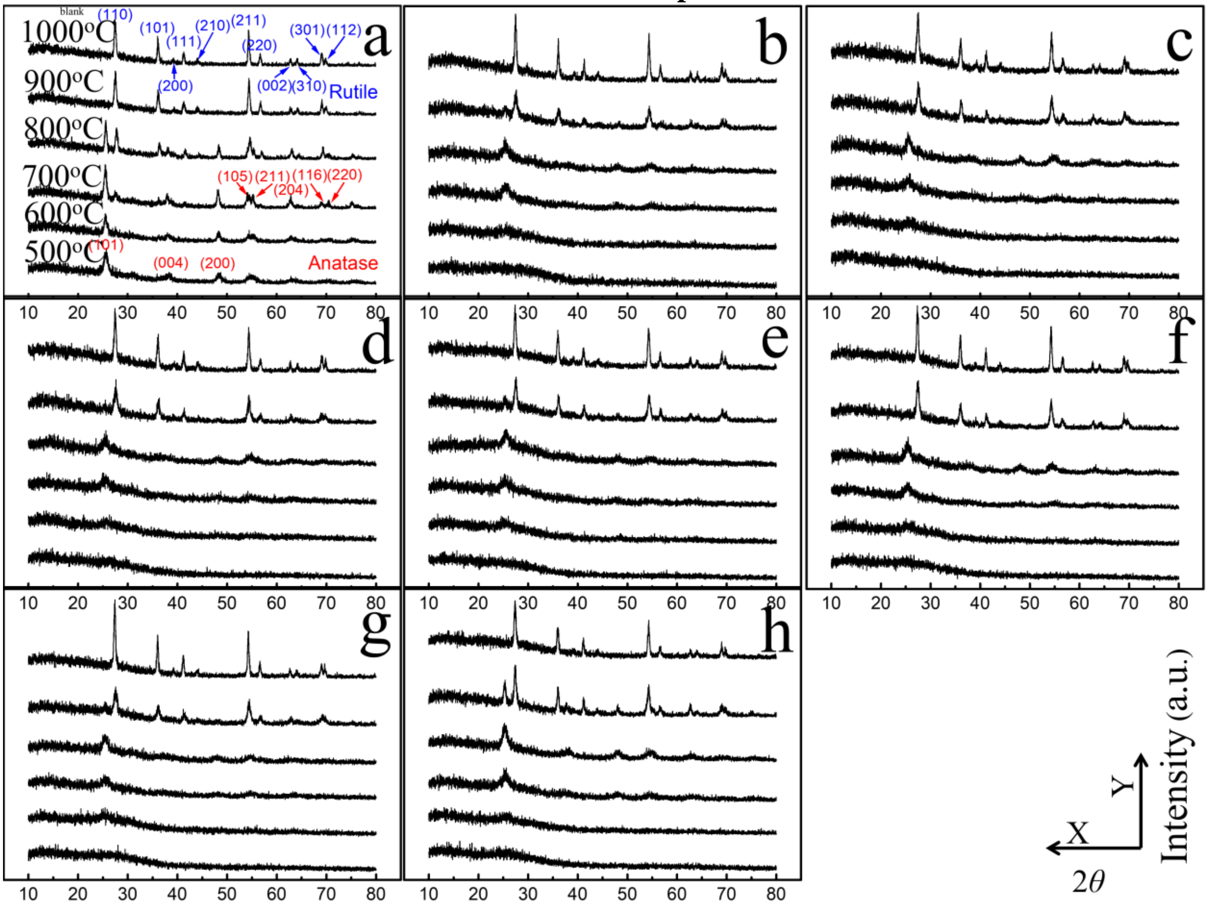

Fig. S7. X-ray diffraction (XRD) patterns of TiO_2-SiO_2 shells obtained at different calcination temperature (500-1000 °C). a) Uncoated PS-TiO_2 particles, and b) to h) corresponds to TPM

coated PS-TiO_2 sample shown in Fig. 2b-1 to 2b-7, respectively. X- and Y-axes are the 2θ and intensity (a.u.) as demonstrated at the lower right corner, respectively.

Table S2. A summary of TiO_2 crystal form and grain size from X-ray diffraction spectra in Fig. S7 as a function of calcination temperature.

Batch no.[a)]	500 °C (nm)	600 °C (nm)	700 °C (nm)	800 °C (nm)	900 °C (nm)	1000 °C (nm)
TiO_2	9.0	10	12.1/14.3[b)]	15.5/18.7	19.1	22.6
2b-1	-	1.9	2.5	7	10/12.9	23.1
2b-2	-	2.7	4.8	6.1	16/15.9	20.4
2b-3	-	2.1	2.6	6.4	14.1	20.4
2b-4	-	2.0	3.5	5.6	10.9/14.8	19.7
2b-5	-	2.4	3.5	7.7	15.4	21.8
2b-6	-	1.6	4	6.8	10/12.1	22.2
2b-7	1.2	1.8	5.9	9.4	17.4/19.4	24.1

[a)] The batch no. corresponds to the sample number in Fig. 2, [b)] the blacks and reds denote the grain sizes of anatase and rutile crystals, respectively.

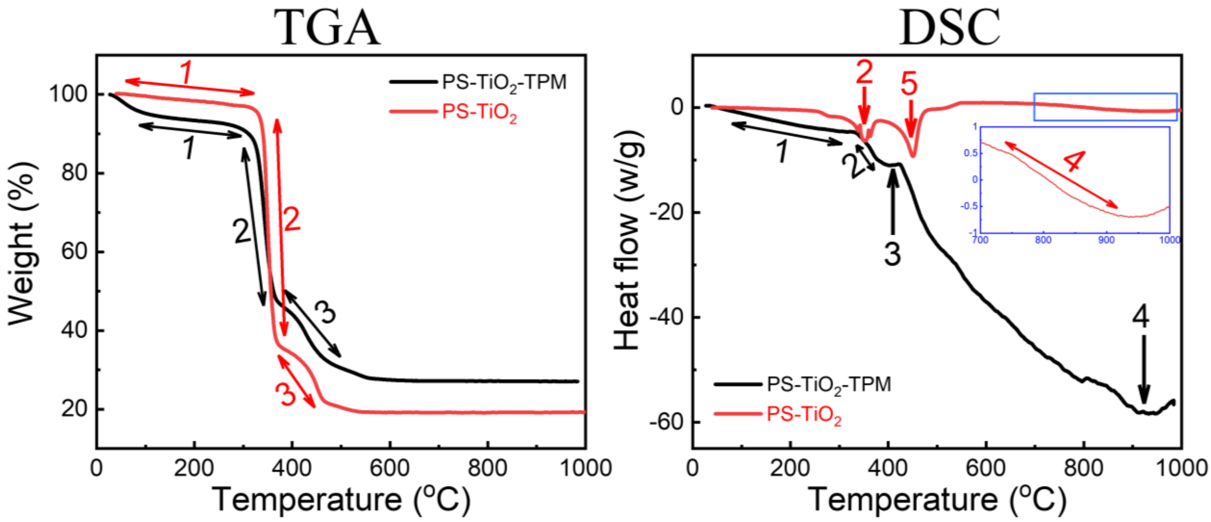

Fig. S8. Thermogravimetric analysis (TGA) and differential scanning calorimetry (DSC) results of PS-TiO_2-TPM and PS-TiO_2 particles measured about 0 to 1000 °C. TGA result: stage 1, low boiling point chemical residual loss; stage 2, decomposition of PS; stage 3, decomposition of TPM and PVP; DSC results: stage 1, the endothermic process corresponding to the evaporation of low boiling point solvents; stage 2, the endothermic peak corresponds to the decomposition of the PS; stage 3, the endothermic peak maybe related to the decomposition of TPM; stage 4, the endothermic peaks at 600-900 and 900 °C for PS-TiO_2 and PS-TiO_2-TPM particles, respectively, refer to the anatase to rutile transition of TiO_2, which indicate the crucial role of TPM lobes in stabilising the anatase crystal phase; stage 5, the endothermic peak at 450 °C may refer to the decomposition of PVP.

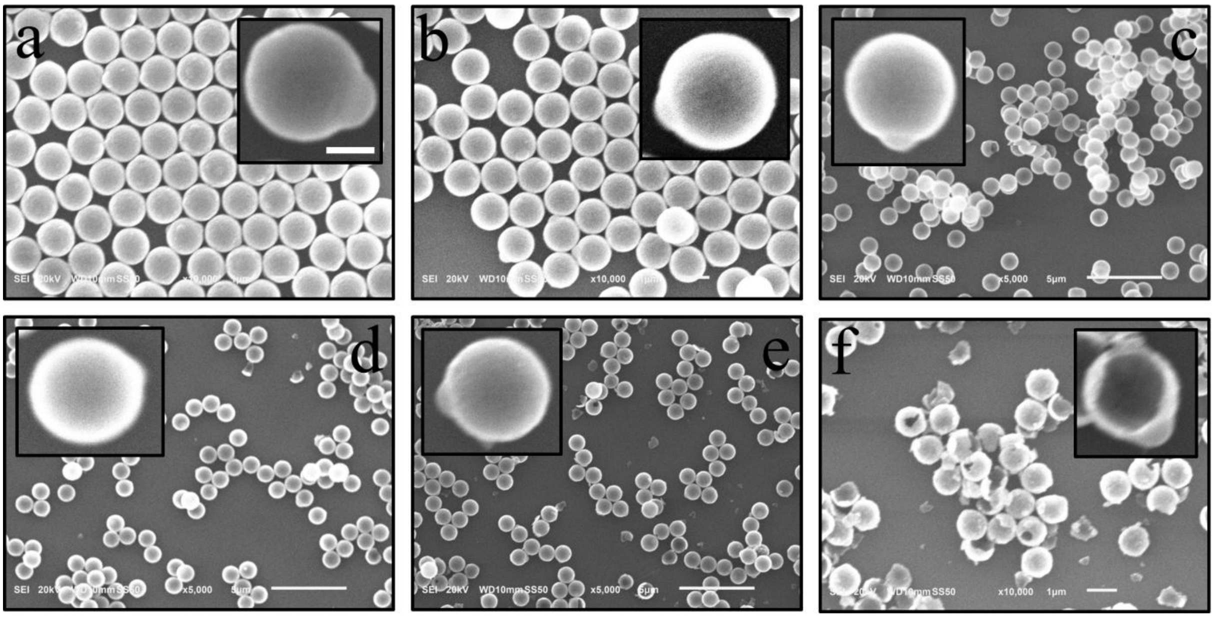

Fig. S9. Raspberry c-TiO_2-SiO_2 particles (Fig. 2b-1 and S7b) calcinated at a) 500, b) 600, c) 700, d) 800, e) 900, and f) 1000 ^{o}C, respectively. The scale bar is 500 nm in the insets.

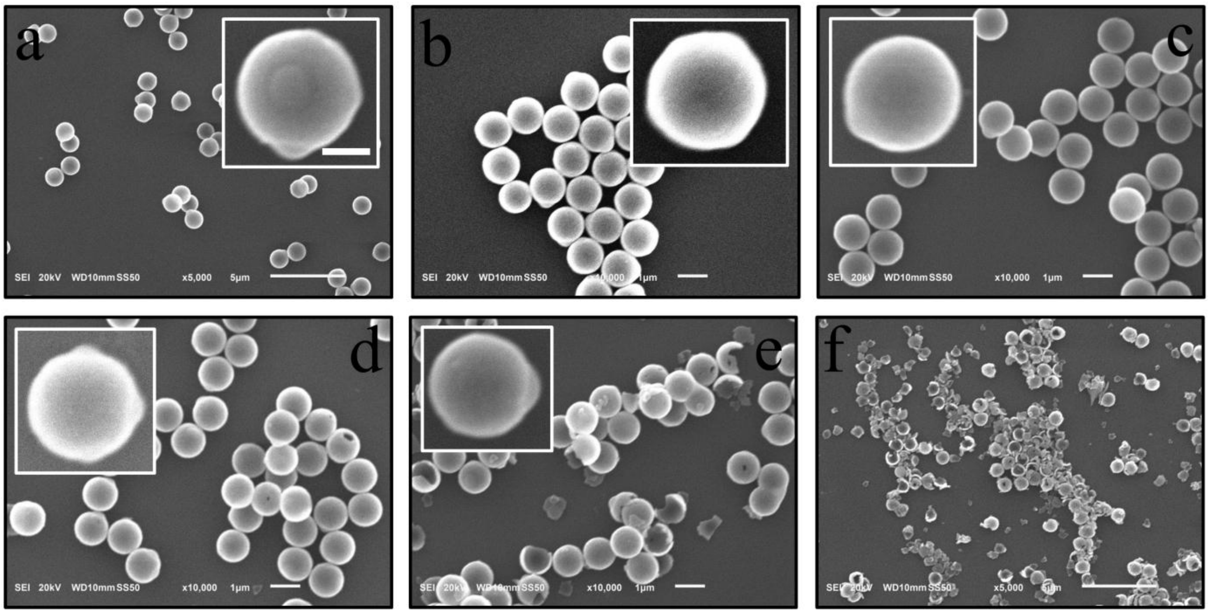

Fig. S10. Raspberry c-TiO_2-SiO_2 particles (Fig. 2b-2 and S7c) calcinated at a) 500, b) 600, c) 700, d) 800, e) 900, and f) 1000 ^{o}C, respectively. The scale bar is 500 nm in the insets.

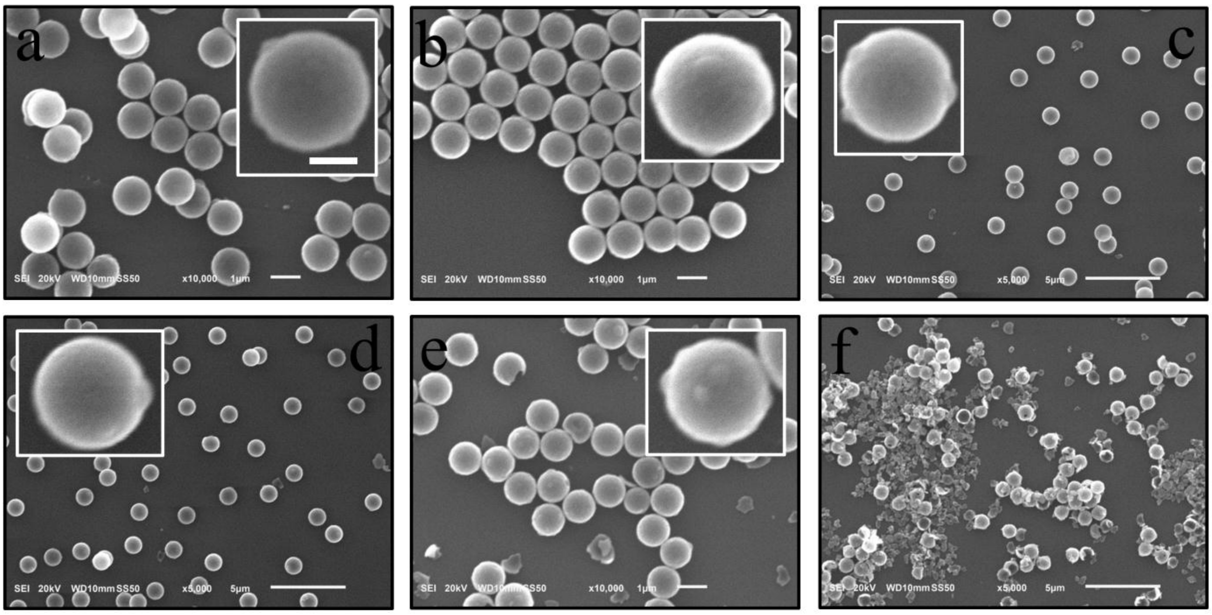

Fig. S11. Raspberry c-TiO_2-SiO_2 particles (Fig. 2b-3 and S6d) calcinated at a) 500, b) 600, c) 700, d) 800, e) 900, and f) 1000 ^oC, respectively. The scale bar is 500 nm in the insets.

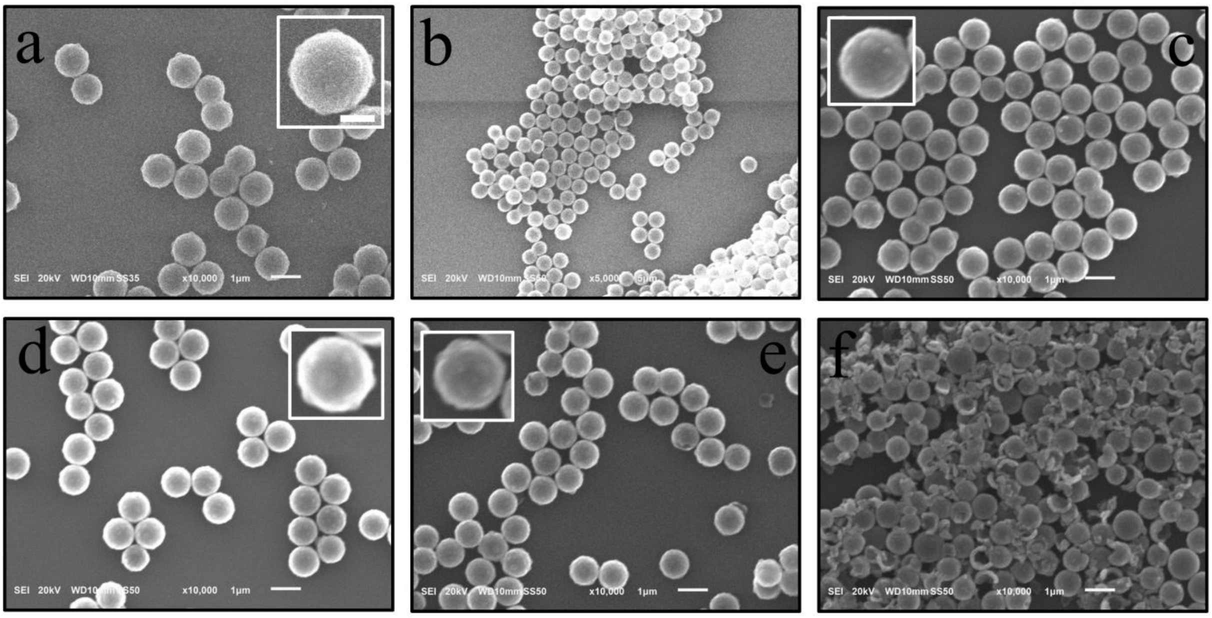

Fig. S12. Raspberry c-TiO_2-SiO_2 particles (Fig. 2b-4 and S7e) calcinated at a) 500, b) 600, c) 700, d) 800, e) 900, and f) 1000 ^oC, respectively. The scale bar is 500 nm in the insets.

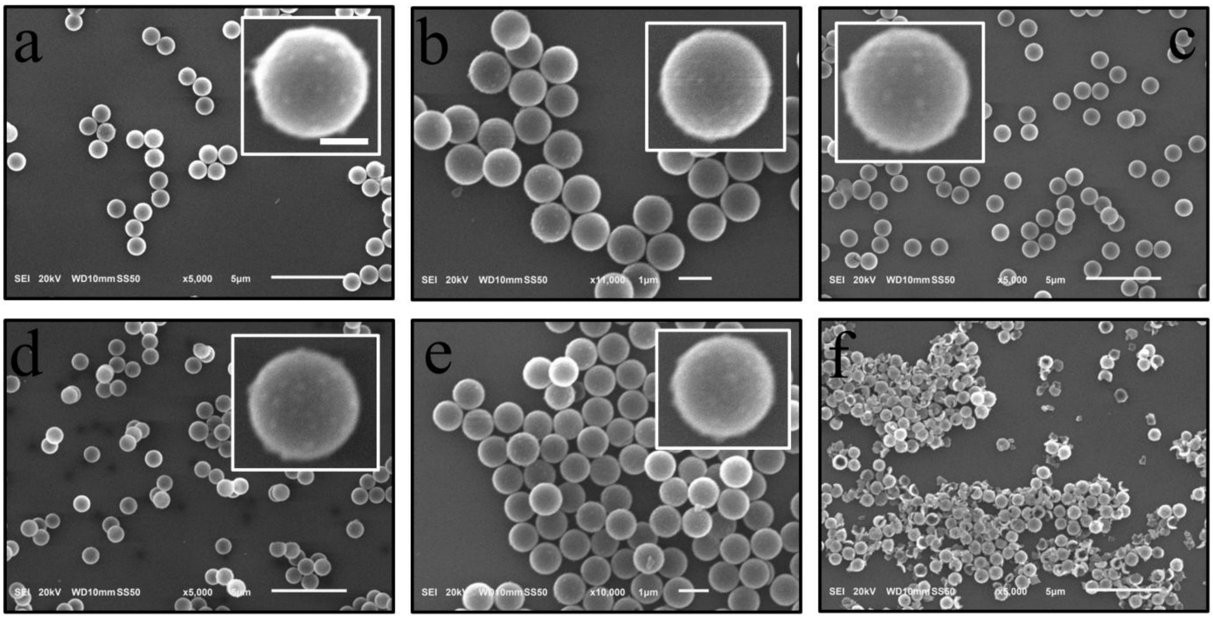

Fig. S13. Raspberry c-TiO_2-SiO_2 particles (Fig. 2b-5 and S7f) calcinated at a) 500, b) 600, c) 700, d) 800, e) 900, and f) 1000 ^{o}C, respectively. The scale bar is 500 nm in the insets.

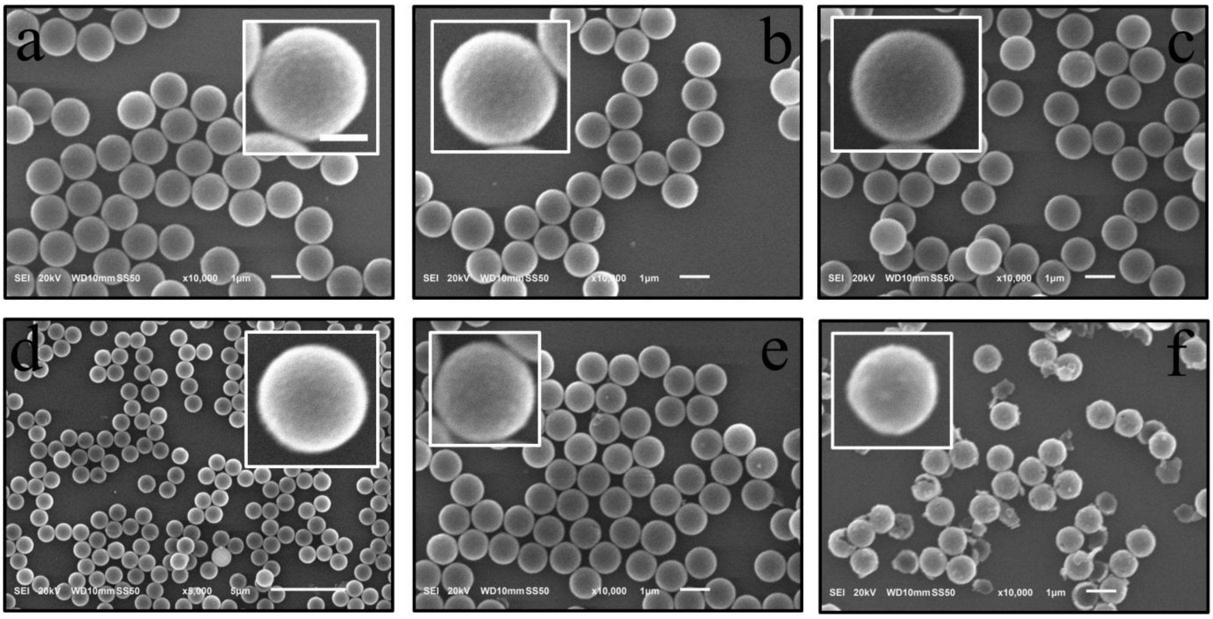

Fig. S14. Raspberry c-TiO_2-SiO_2 particles (Fig. 2b-6 and S7g) calcinated at a) 500, b) 600, c) 700, d) 800, e) 900, and f) 1000 ^{o}C, respectively. The scale bar is 500 nm in the insets.

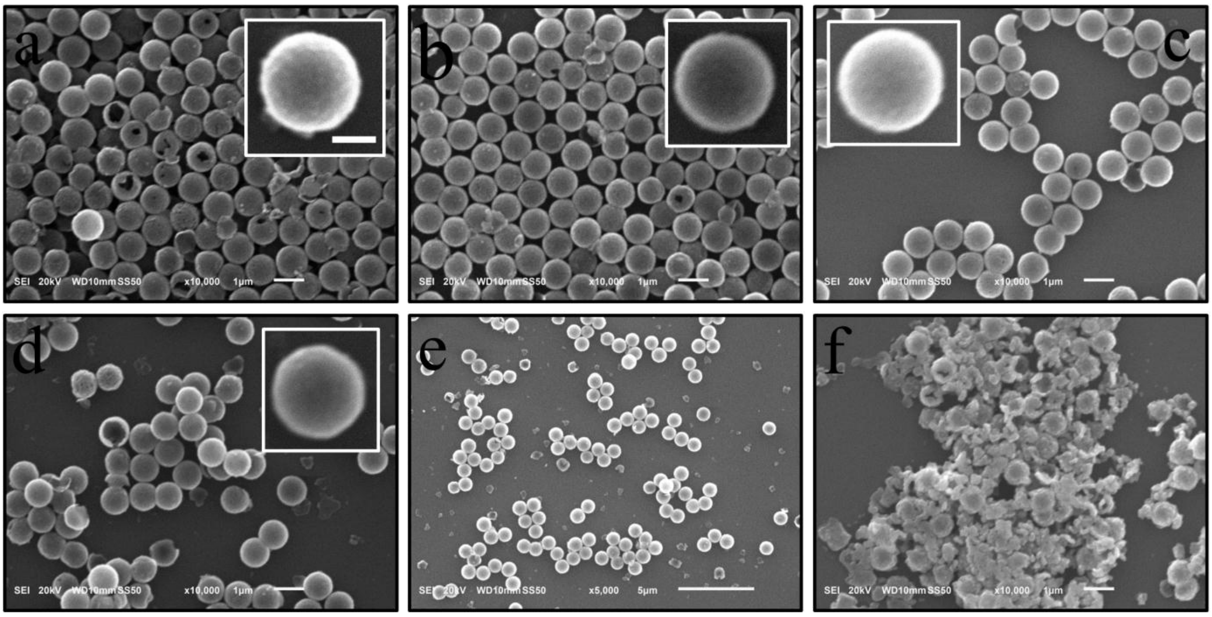

Fig. S15. Raspberry c-TiO_2-SiO_2 particles (Fig. 2b-7 and S7h) calcinated at a) 500, b) 600, c) 700, d) 800, e) 900, and f) 1000 ^oC, respectively. The scale bar is 500 nm in the insets.

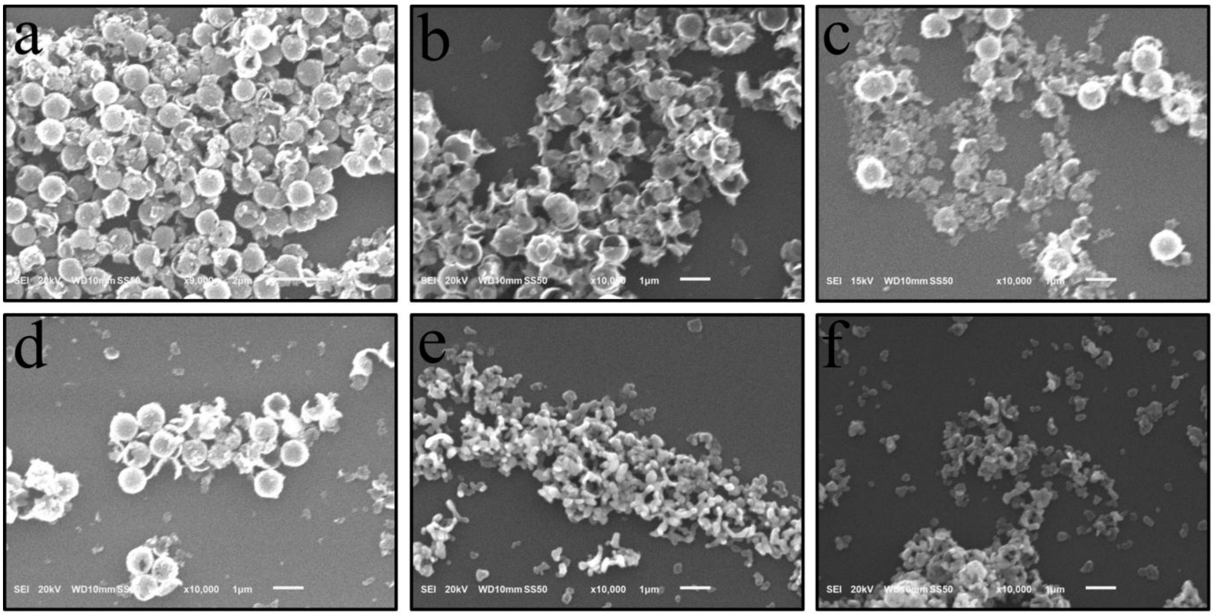

Fig. S16. SEM images of uncoated pristine TiO_2 shells (Fig. 1c and S7a) calcinated at a) 500, b) 600, c) 700, d) 800, e) 900, and f) 1000 ^oC, respectively. The scale bars are 1 μm. Pristine TiO_2 shells cannot maintain their hollow structure during the calcination in the absence of TPM protection.

1.3 Photo-activity Characterization of Coated and Uncoated TiO_2 Shells

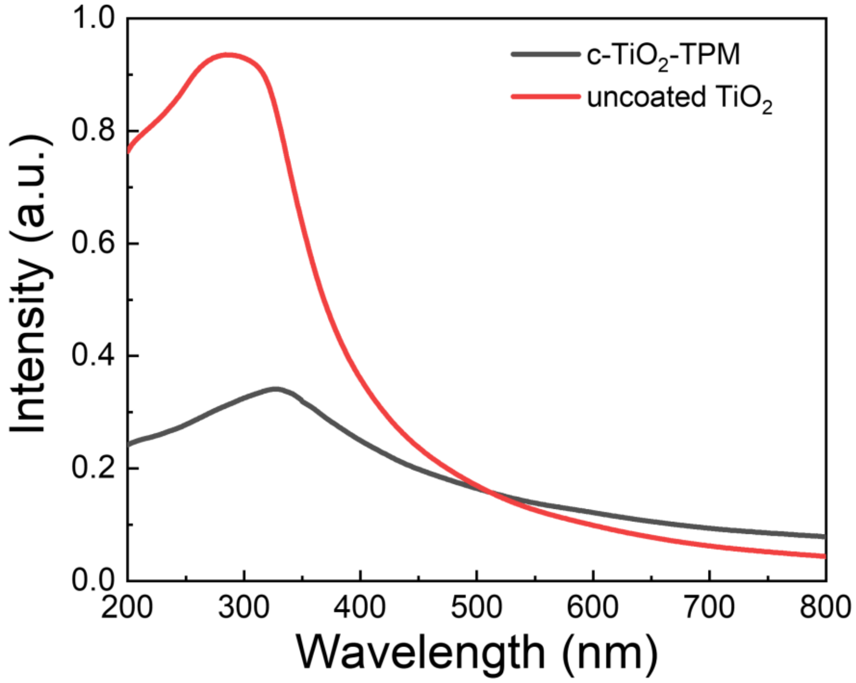

Fig. S17. UV-Vis absorption of coated and uncoated TiO_2 shells. The absorption peak of uncoated TiO_2 is about 290 nm, which is consistent with the absorption of anatase TiO_2 nanocrystals.[1] In contrast, the c-TiO_2-SiO_2 shells exhibit a broad absorption peak at around 310 nm, which may be caused by the SiO_2 nodules.[2]

1.4 Morphology Characterization of Uncoated TiO_2 Shells

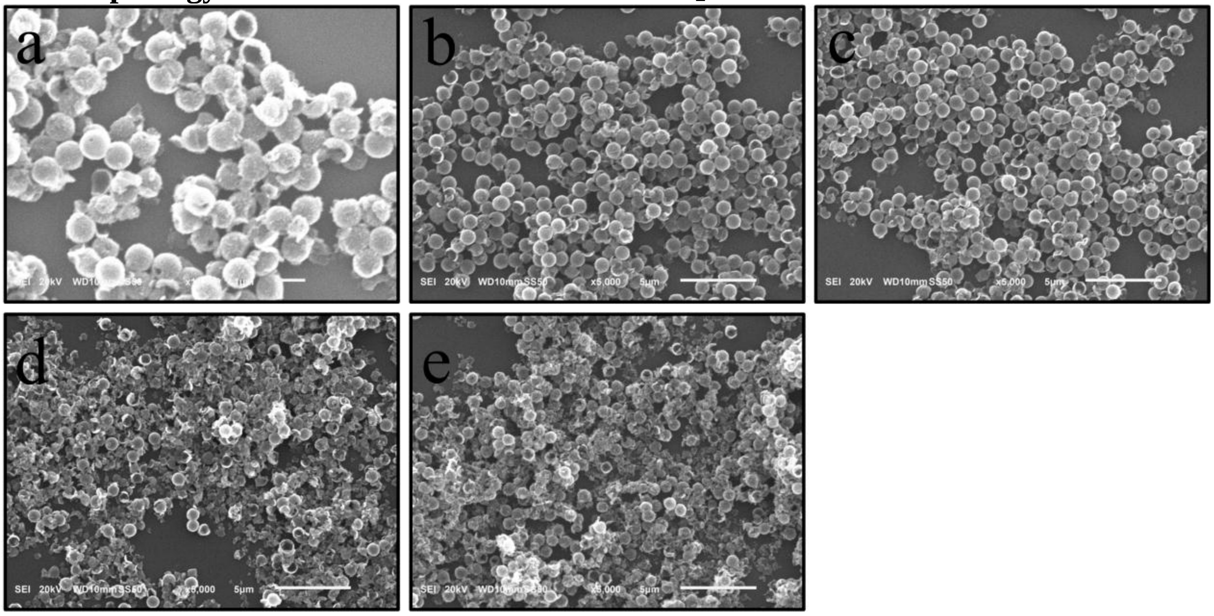

Fig. S18. Uncoated TiO_2 shells that were obtained by removing the SiO_2 lobes of c-TiO_2-SiO_2 shells with NaOH after the calcination at a) 600, b) 700, c) 800, d) 900, and e) 1000 ^{o}C.

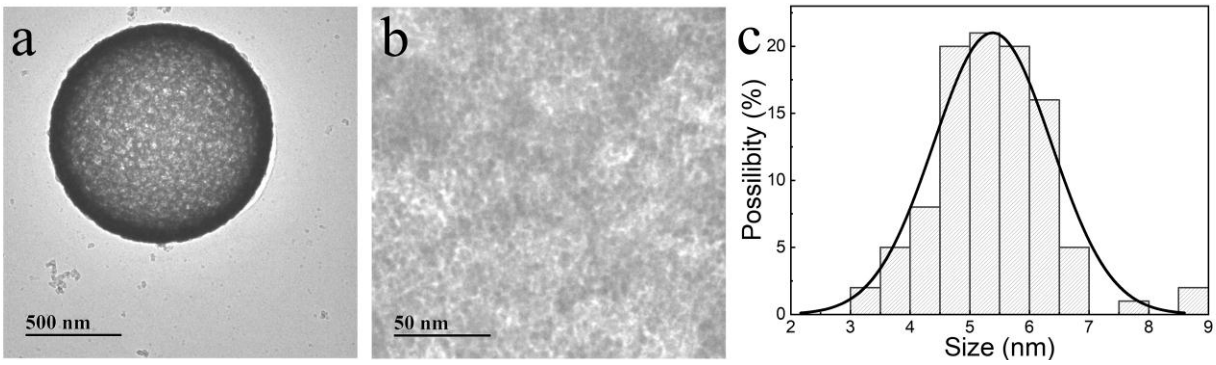

Fig. S19. a) and b) TEM observations of uncoated TiO_2 shells that were obtained by removing the SiO_2 lobes of c-TiO_2-SiO_2 shells with NaOH after the calcination at 800 ^oC. c) The grain size distribution corresponding to the TEM image in b) exhibits a mean grain size of 5.38 nm, which is slightly smaller than the grain size of TiO_2 shells prior to the NaOH treatment (c.f. Fig. S3g). This decrease may be ascribed to the dissolution of silicate between grains.

1.5 Stability Evaluation of Pristine, Coated, and Uncoated TiO_2 Shells after Photocatalysis

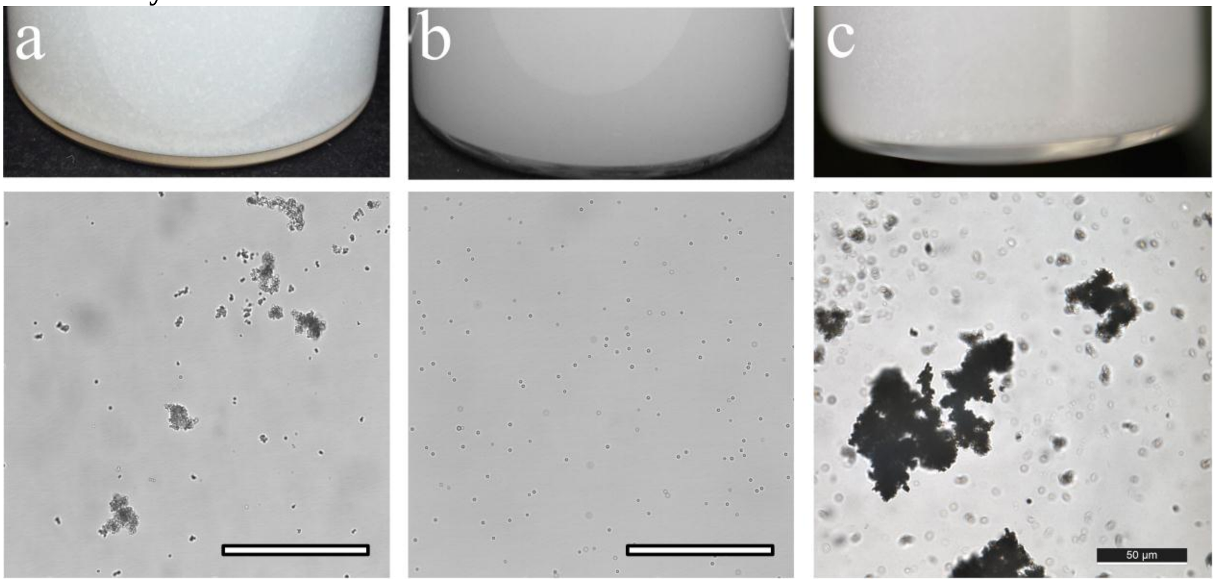

Fig. S20. Optical micrographs of a) uncoated TiO_2 shells (i.e., the same particles as in b) but *after* the removal of SiO_2 with NaOH) and b) coated TiO_2 shells (c-TiO_2-SiO_2, sample in Fig. S13d), and c) pristine TiO_2 shells after photocatalysis. The scale bar is 50 μm. The uncoated and pristine TiO_2 shells tends to aggregate during the photocatalysis, while the c-TiO_2-SiO_2 particles are stable.

1.6 Photocatalytic Activity of Pristine TiO_2 Shells

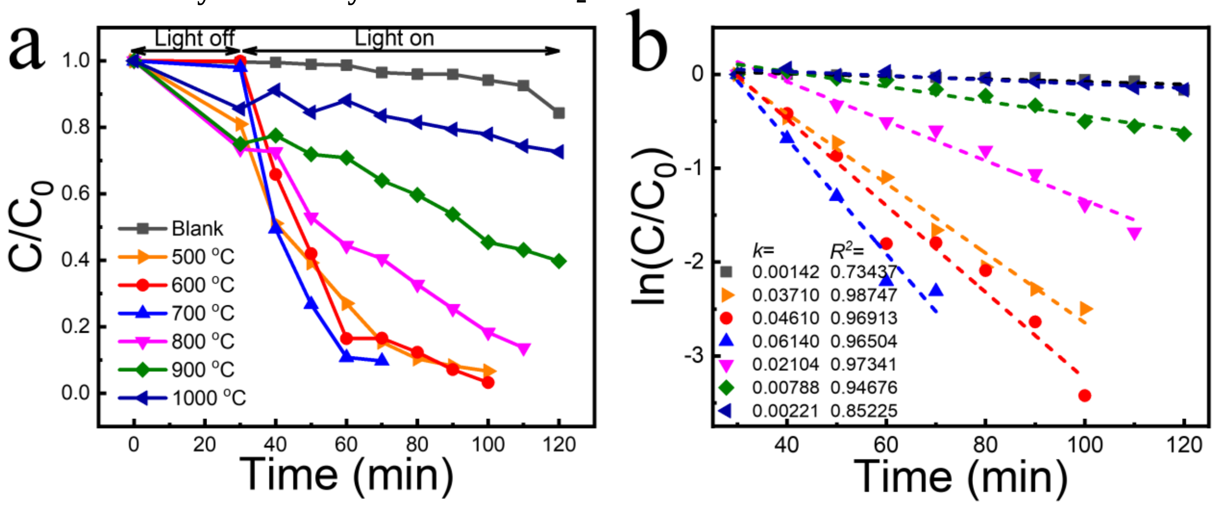

Fig. S21. The photocatalytic activity evaluation of pristine TiO_2 shells during the degradation of rhodamine b isothiocyanate (RITC) under UV irradiation measured at the wavelength of maximum absorbance of RITC. a) The relative absorbance (C/C_0) as a function of the irradiation time using pristine TiO_2 shells calcinated at temperatures between 500 to 1000 ^oC; b) the corresponding fits to the data in a) to obtain the effective first order reaction rate constants (*k*).

2. Supplementary References:

[1] H. Ceylan, C. Ozgit-Akgun, T. S. Erkal, I. Donmez, R. Garifullin, A. B. Tekinay, H. Usta, N. Biyikli, M. O. Guler, *Sci. Rep.*, **2013**, *3*, 2306.

[2] A. Jaroenworaluck, N. Pijarn, N. Kosachan, R. Stevens, *Chem. Eng. J.*, **2012**, *181-182*, 45.
